# Supplementary material for: Deletion of LBR N-terminal domains recapitulates Pelger-Huet anomaly phenotypes in mouse without disrupting X chromosome inactivation
Source: Commun Biol. 2021 Apr 12;4:478. doi: 10.1038/s42003-021-01944-2 (PMC8041748; doi:10.1038/s42003-021-01944-2)
Supplement: Supplementary file 2 — Supplementary Information [file 42003_2021_1944_MOESM2_ESM.pdf]

Supplementary Figure 1: *Generation and characterization of Lbr 236 mutation*

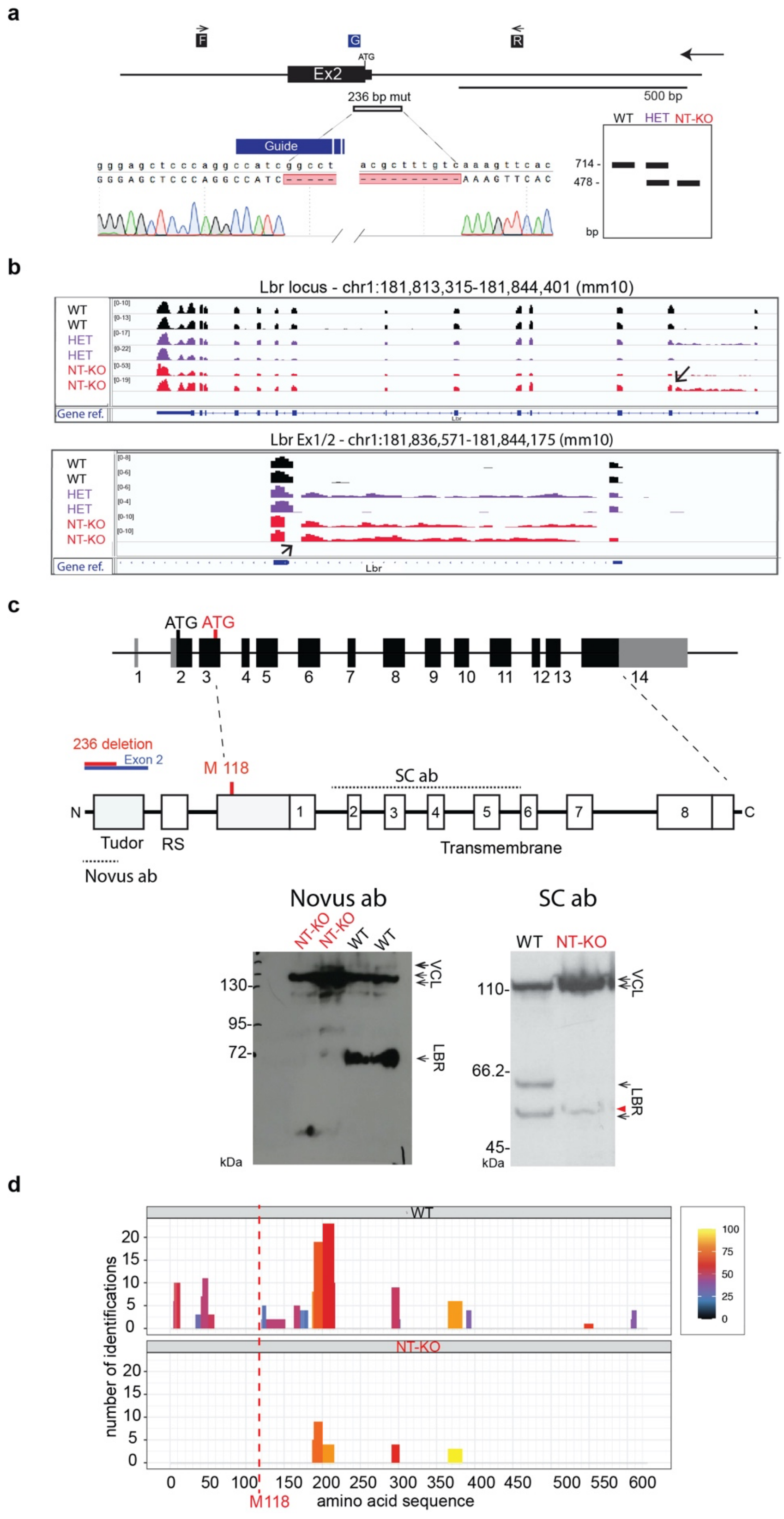

a) Top, schematic representation of the studied mutation. Genomic features, the position of the CRISPR/Cas9 guides, and a Sanger sequencing panel are shown. Bottom right, the schematic PCR-based genotyping approach. **b)** RNA-seq analysis in *Lbr* WT, Heterozygous (HET) and *Lbr* N-term KO (NT-KO) tissues (liver), reveals aberrant *Lbr* intron 1 transcription; site of mutation is shown (black arrow). **c)** Top, Schematic of *Lbr* gene locus and protein product indicating hypothesised alternative translation start site (M118) of the 236bp mutant. Domains and the relevant exon/mutation (blue, red respectively) are shown. Bottom left, the Novus ab only detects a specific protein product in the WT lanes. Right, LBR WB (SC ab) analysis showing the truncated allele described in the text (red triangle). An antibody recognising the central region of LBR (SC ab, Santa Cruz antibody, the exact position is not known) and one recognising the N-terminal part (Novus, LBR aa 1-70), have been used. The WB analysis shows a pattern that is compatible with the computer prediction of the alternative translation start site usage (M118 WT protein). Note: The lower band in the WT is likely to be a N-term degradation product running at a similar molecular weight than our LBR NT-KO protein (aa 118-626, red triangle). Vinculin (VCL) has been used as a loading control **d)** Mass-Spectrometry analysis of the LBR IPs supporting the conclusion of an N-terminal truncation (no N-term peptides were recovered; 2 duplicates). Number of peptides recovered and their position is shown. A colour-coded legend in the figures, indicate the confidence of peptide detection.

Supplementary Figure 2: *Lbr 236 homozygous mutant mice do not show evident defects in gametes*

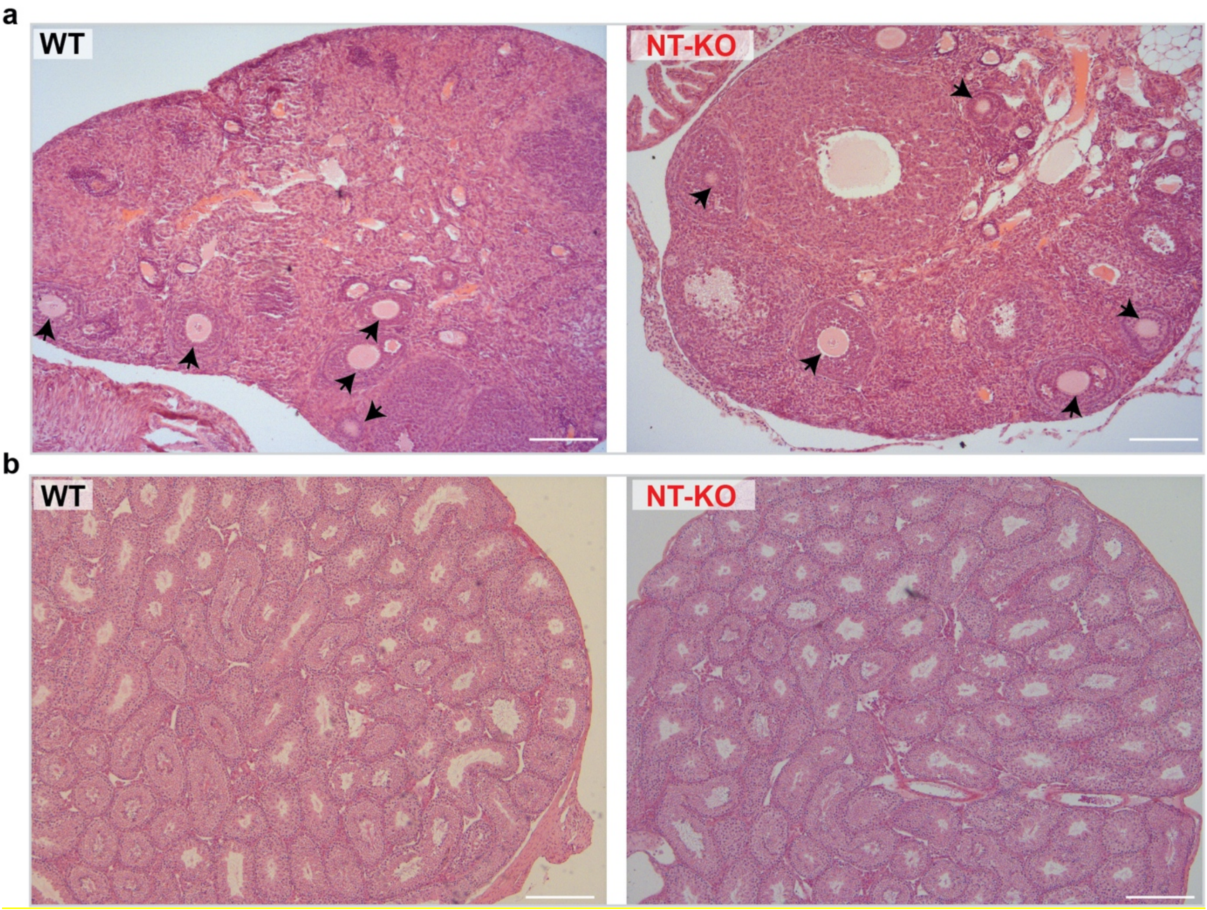

a) Ovaries are shown from WT and NT-KO animals. Eggs are indicated by black arrows. **b**) Testicles sections are shown from WT and NT-KO animals. No visible defects can be detected. Scale bar indicates 300 µm.

Supplementary Figure 3: GO *analysis of WT and NT-KO animals*

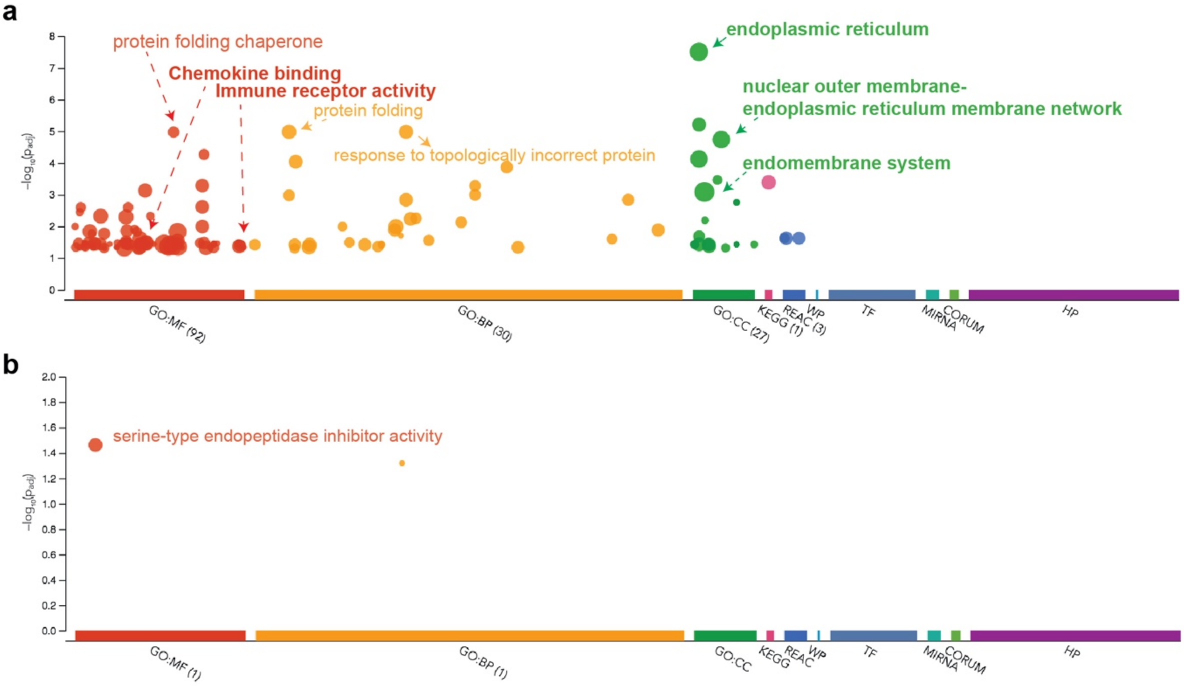

a) Analysis in male NT-KO vs WT is shown (liver). **b)** Analysis in female NT-KO vs WT is shown. The biological processes (BP), molecular functions (MF), cellular components (CC) are shown.

Supplementary Figure 4: ***An equal number of cells initiate XCI in Lbr WT and NT-KO female ES cells***

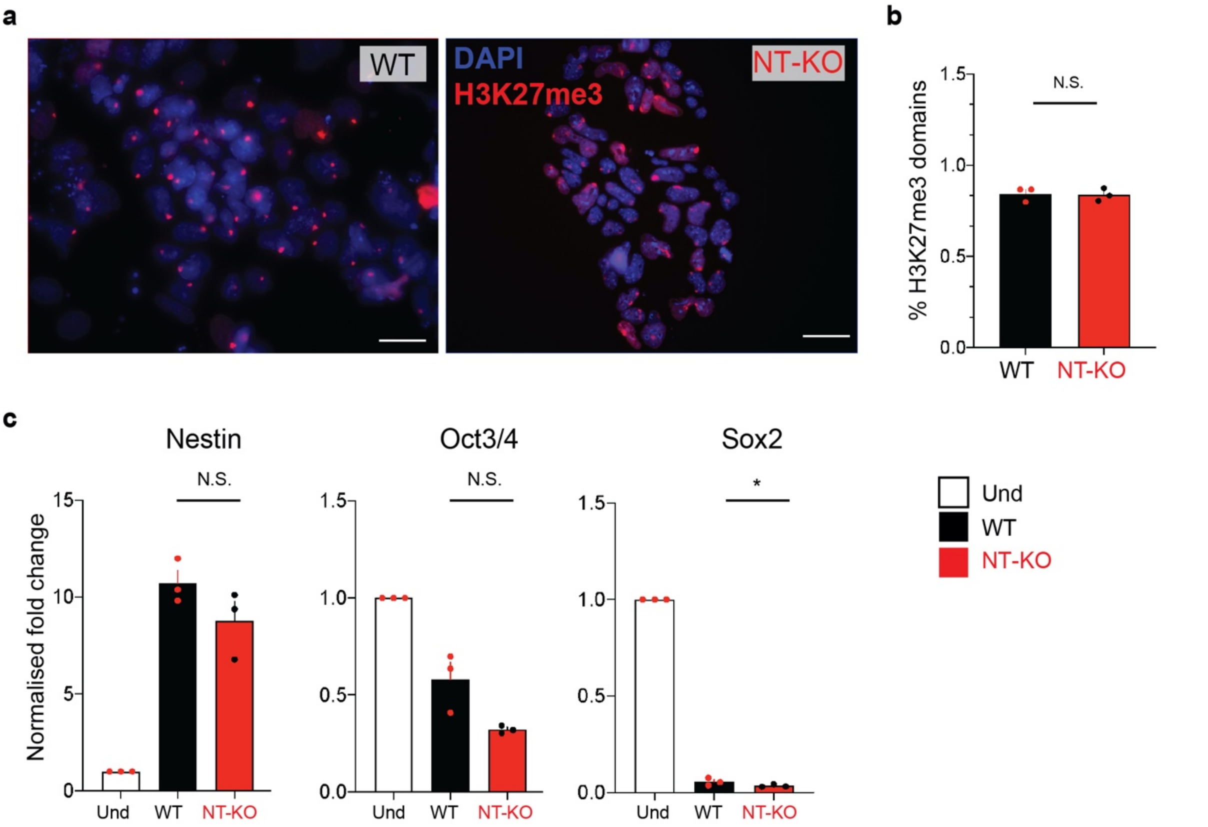

a) H3K27me3 IF analysis of WT and NT-KO, representative images are shown. **b)** H3K27me3 Immunofluorescence analysis show no difference in the number of cells starting XCI at day 5 of differentiation (n=598). H3K27me3, red; DAPI, blue. Bars indicate 40µm. **c)** qRT-PCR analysis of differentiation markers. Nestin, Oct3/4, Sox2 are shown. Samples are indicated in the legend. Data from 3 biological samples are shown. * Indicates statistical significant 2-tailed t-test ($p \leq 0.05$). p-value: Sox2 WT/NT-KO, $p=0.0425$. Gapdh has been used as internal normalization control. Samples have been normalized to the undifferentiated condition (Und), to show efficient and comparable differentiation.

Supplementary Figure 5: *Unprocessed Western Blots (WB) replicas and an example of PCR genotyping*

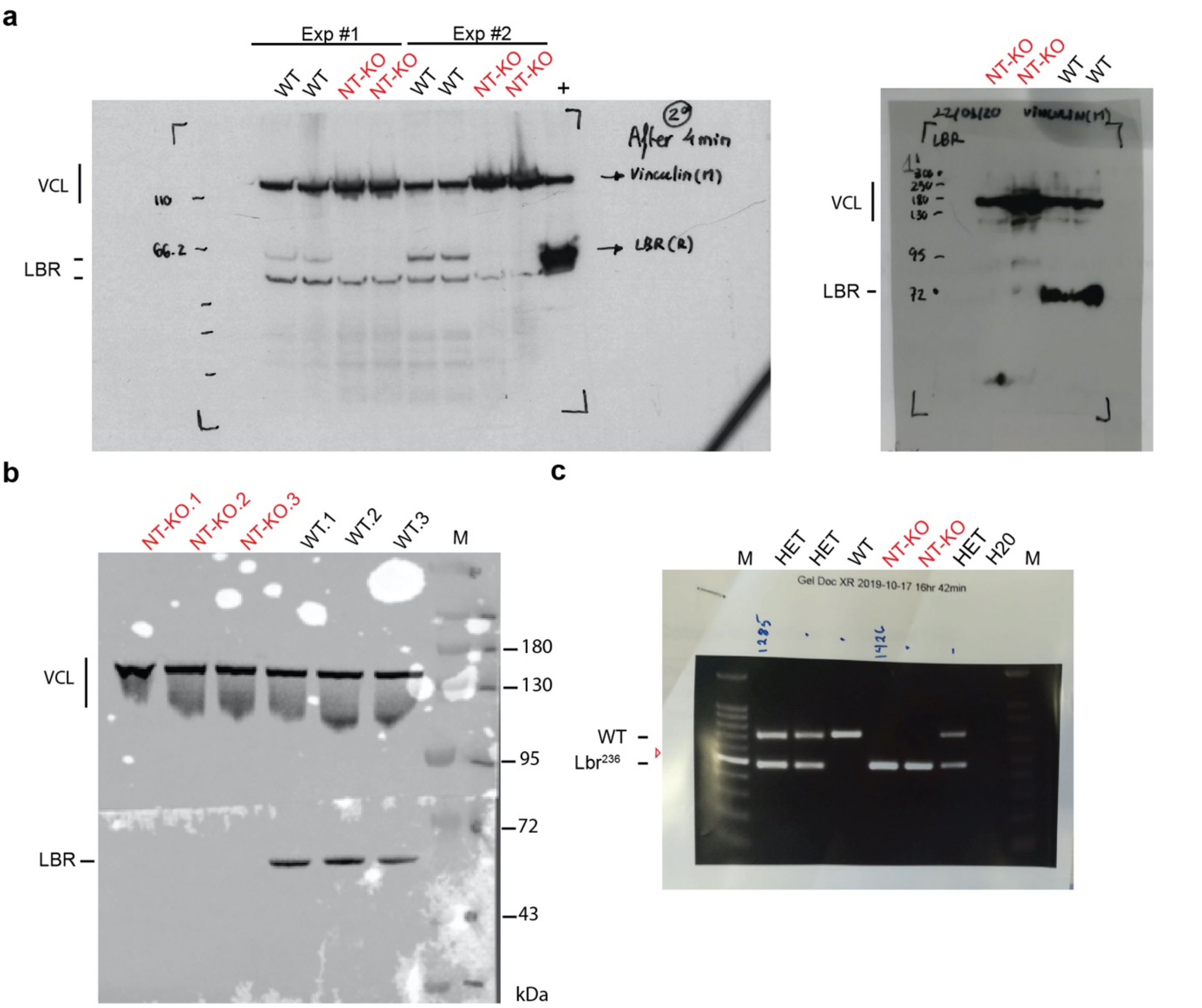

a) Left, unprocessed LBR Western Blots using the Santa Cruz antibody (see **Fig. S1**, Material and Methods). Two independent experiments in biological replicates are shown. Sample IDs are shown (Wild-Type (WT) and LBR N-term KO (NT-KO); +: purified LBR protein. Right, unprocessed LBR Western Blots using the Novus Biological antibody (see **Fig. S1**, Material and Methods). Two technical replicates are shown. Vinculin (VCL) has been used as a loading control**.** LBR, is also shown (LBR). Sample IDs are shown (Wild-Type (WT) and Lbr N-term KO (NT-KO). **b)** Unprocessed replica of the Novus Biologicals antibody. Three biological replicates are shown. Vinculin (VCL) has been used as a normalization control**.** LBR, is also shown (LBR). Samples IDs are shown (Wild-Type (WT) and LBR N-term KO (NT-KO), M indicates the protein marker. **c)** A representative image of the PCR genotyping is shown. Top band (714bp), WT; lower band (478bp), *Lbr*^{236} mutant.1Kb+ marker and the H2O negative control are also shown, red triangle indicates the marker's 500bp band. Mice genotypes (Wild-Type, WT; heterozygous, HET; Homozygous mutants, NT-KO) are shown on top of each lane.
