# Supplementary material for: Deletion of LBR N-terminal domains recapitulates Pelger-Huet anomaly phenotypes in mouse without disrupting X chromosome inactivation
Source: Commun Biol. 2021 Apr 12;4:478. doi: 10.1038/s42003-021-01944-2 (PMC8041748; doi:10.1038/s42003-021-01944-2)
Supplement: Supplementary file 3 — Description of Additional Supplementary Files [file 42003_2021_1944_MOESM3_ESM.pdf]

Description of Additional Supplementary Files

File name: Supplementary Data 1

Description: Processed RNA-seq results for KO vs WT mice. Results are shown separately for male and female mice.

File name: Supplementary Data 2

Description: Source data for the main and supplementary figures.

File name: Supplementary Data 3

Description: Primers used in this study.
